# Analysis of Carcass and Meat Characteristics in Breast Muscle Between Hubbard White Broilers and Xueshan Chickens

**DOI:** 10.3390/ani15142099

**Published:** 2025-07-16

**Authors:** Fan Li, Xingyu Zhang, Jiajia Yu, Jiaxue Yuan, Yuanfeng Zhang, Huiting He, Qing Ma, Yinglin Lu, Xiaoe Xiang, Minli Yu

**Affiliations:** Department of Animal Genetics, Breeding and Reproduction, College of Animal Science and Technology, Nanjing Agricultural University, Nanjing 210095, China; 2022805140@stu.njau.edu.cn (F.L.); 2023105039@stu.njau.edu.cn (X.Z.); 15123401@stu.njau.edu.cn (J.Y.); yuanjiaxue@stu.njau.edu.cn (J.Y.); 15123416@stu.njau.edu.cn (Y.Z.); 2024105040@stu.njau.edu.cn (H.H.); 2024805156@stu.njau.edu.cn (Q.M.); luyinglin@njau.edu.cn (Y.L.); xxxiang@njau.edu.cn (X.X.)

**Keywords:** broiler, carcass trait, meat quality, lipid metabolism gene, native breed

## Abstract

The focus on selecting broilers for rapid growth rates and enhanced breast muscle yield has resulted in a decline in meat quality. The differences in carcass characteristics and meat quality between Hubbard white broilers (HWs, a commercial breed) and Xueshan chickens (XSs, an indigenous breed) at market age were analyzed to determine the potential mechanisms responsible for these differences. The results show that HWs exhibited significantly better carcass performance than XSs, including the larger weight of the carcass, the breast muscle, and the thigh muscle (*p* < 0.01). In addition, based on HE staining analysis, HWs’ breast muscles had a considerably larger average myofiber area and diameter than those of XSs (*p* < 0.01). Furthermore, the physical characteristics of the meat revealed that XSs had higher redness and yellowness and also higher lightness. HW meat had a higher pH and thermal loss, but a lower shear force and drip loss than XS meat (*p* < 0.01). The content of saturated fatty acids (SFAs) and polyunsaturated fatty acids (PUFAs) was, remarkably, lower in the breast muscles of HWs than of XSs (*p* < 0.01). In contrast, HWs had a larger concentration of monounsaturated fatty acids (MUFAs) than XSs (*p* < 0.01). Finally, the breast muscles of XSs had lower levels of mRNA expression for genes linked to lipid metabolism, such as fatty acid binding protein 4 (*Fabp4*) and peroxisome proliferator-activated receptor alpha (*Pparα*), and had higher levels of the phosphofructokinase muscle type (*Pfkm*) compared to HWs (*p* < 0.01). These results indicate that a lower carcass yield was observed in XSs compared with HWs, but that XSs showed better performance in terms of meat quality than HW.

## Simple Summary

The comparative analysis reveals fundamental differences in muscle biology between commercial and native chicken breeds. While intensive selection in HW enhances growth efficiency and muscle hypertrophy, it concurrently reduces meat quality through an altered fiber architecture and lipid composition. The elevated PUFA content (particularly n-3 fatty acids) in XS suggests greater potential for flavor compound formation during cooking. The distinct post-mortem biochemical profiles, evidenced by differential pH decline rates and protein degradation patterns, indicate breed-specific muscle-to-meat conversion processes. These findings provide actionable insights for developing breeding strategies that balance production efficiency with meat quality preservation.

## 1. Introduction

Currently, the consumption of poultry meat has witnessed a rapid increase, and it is anticipated that this upward trend will persist in the future [1]. However, in order to acquire greater profits, many companies have chosen to raise chickens with fast growth rates and high body weights [2]. This has also led to changes in the quality and flavor of the chicken meat. As living standards continue to rise, consumers increasingly prioritize higher nutritional value and are willing to pay a premium for products that meet their standards.

Hubbard white broilers (HWs), a widely utilized commercial breed, have been selected for their fast growth rate, high meat yields, and low rate of abdominal fat deposition [3]. Nowadays, the HW is one of the main standard broilers in Chinese markets. However, its rapid growth and high yield may have a detrimental impact on both sensory attributes and meat quality [4]. The Xueshan chicken (XS) is a slow-growing chicken breed with three distinct yellow features: feet, beak, and feathers [5]. The majority of consumers prefer native-breed chickens to standard broilers because of their distinct taste and flavor. As in other Asian countries, in China the trend of native chicken production and consumption continues to rise faster than that of commercial broilers. However, the advantages and disadvantages of XSs and commercial breeds in terms of slaughter and meat quality traits remain unclear.

Three primary categories of meat chickens are raised in China: native breeds, commercial broilers, and hybrids [6]. Previous studies have shown that local chicken breeds’ meat quality and sensory attributes are preferred over those of commercial broilers [7,8,9]. Meat quality and carcass traits serve as crucial indicators for evaluating the performance of the poultry industry. Meat quality is a broad concept consisting of multiple traits such as pH, color, texture, water-holding capacity, drip, and cooking loss. There are variations in the meat quality and sensory attributes of breast meat from broilers with different growth rates [5]. Different broiler breeds exhibit differences in muscle metabolic patterns, which result in variations in their flavor profiles [10].

Genotypes that regulate muscle development during growth are the main reason for differences in meat quality among different chicken breeds. It has been found that, under the same external environmental conditions, Wenchang chickens and Xianju chickens had reduced cooking loss and expressible moisture, higher shear value and more fatty acids than Hy-Line Brown layer line chickens [11]. The distinctive characteristics of Ufipa indigenous chicken meat, in terms of nutritional value, texture, and flavor, render it superior to the meat of commercial Ross chickens [12].

This study aimed to compare the performance in terms of carcass traits, meat quality, and the potential molecular mechanisms between HWs and XSs. In this study, the histological trait of muscle fiber, the content of amino acids and fatty acids, and the expression level of lipid metabolism-related genes in the breast muscles at market ages were measured. These results will provide insights into the factors affecting meat quality and give consumers information pertinent to choosing between commercial breeds or local chickens.

## 2. Materials and Methods

### 2.1. Ethics Statement

The Laboratory Animal Welfare and Ethics Committee of Nanjing Agricultural University, China (Permit number: SYXK-2021-0086), approved this study.

### 2.2. Animals and Sample Collection

A total of 40 male chickens were sampled: slow-growing native chickens (13 weeks old for Xueshan chickens, *n* = 20) and fast-growing commercial breeds (6 weeks old for Hubbard white broilers, n = 20, respectively). The chickens were fed the same complete compound under the same feeding conditions. The chickens were fasted for 12 h with free access to water before slaughter. The chickens were slaughtered according to the relevant regulations applied in the poultry industry. The birds of each group were electrically stunned in a water bath (240 mA, 120 V, 5 s), and were soft-scalded at 53 °C for 120 s. The abdominal fat, breast, and thigh muscle were meticulously dissected, weighed, and the percentage of each part was calculated, respectively.

### 2.3. Hematoxylin and Eosin Staining

Six breast muscle samples were collected from each breed. These samples were first rinsed thoroughly with saline to remove any debris and then fixed in 4% paraformaldehyde for 24 h. Subsequently, the fixed samples were processed for paraffin embedding. Using a microtome (Leica Biosystems, Wetzlar, Germany), the paraffin-embedded samples were sectioned into slices with a thickness of 5 μm. Five non-intersecting fields were randomly at random for image capture for every sample with the use of a microscope (Olympus BX50, Tokyo, Japan). The myofiber cross-sectional area (CSA) and myofiber diameter (MFD) were analyzed by utilizing Image-Pro Plus 6.0 software.

### 2.4. Physicochemical Analysis

The physicochemical properties of the broilers were analyzed by taking samples of their breast muscles. To assess the meat quality, the color of the breast muscles was measured at 24 h post-mortem using a Minolta CR 310 Chroma Meter (Konica Minolta Sensing Business Unit, Osaka, Japan). The device was calibrated using a white and black calibration plate as in a previous study [13], which were graded for L* (brightness), a* (red), and b* (yellow) indices.

The handheld pH meter (Model PC 510, Cyber scan, Singapore) was adjusted according to buffers (pH 4.01 and 7.00). The pH level of the breast muscles was determined at 1 h post slaughter (pH_1_). The test samples were stored in a refrigerator at 4 °C, and the pH reading was taken once again 24 h later (pH_24_). The measurement of the pH was conducted according to a previous study [14].

To calculate cooking losses, each sample—that is, half of a breast—was weighed as weight 1 (W1) and placed in a boiling bag. The samples were placed in a water bath the temperature of 80 °C until the internal temperature of the pieces reached 75 °C, according to the previous study [15]. The samples were subsequently reweighed (W2) after cooling to room temperature.

The water-holding capacity (WHC) of the meat was estimated by the filter paper press method [16]. A sample weight of about 1 g was weighed (W3), and it was placed between double-sided 16-layer filter sheets. Subsequently, the sample was pressurized with 343 N for a period of 5 min, then the weight was taken once again (W4). The WHC of the meat was calculated based on the percentage of water lost from the meat.

The drip loss was calculated as the ratio of the decrease in weight before and after storage at a low temperature. The breast muscle samples were placed in a sealable plastic bag and kept at 4 °C for 24 h. After gently blotting the surface of the meat samples with clean filter paper to remove any excess juice, the samples were then re-weighed.

Tenderness was evaluated by shear force analysis conducted by a testing machine (Model 5542, Instron Engineering Corp, Norwood, MA, USA). The samples used for the determination of cooking loss were cut parallel to the muscle fiber direction by a 250 kg load cell and with a cross-head speed of 500 mm/min. Each sample was measured five times, and the average for each sample was calculated.

### 2.5. Amino Acid Composition

The amino acid profile was measured as previously described [17]. After drying and crushing the meat samples, the L-8900 model automatic amino acid analyzer (Hitachi, Tokyo, Japan) was used to determine the content and composition of the amino acids in the breast muscles.

### 2.6. Fatty Acid Composition

The fatty acid (FA) composition of the samples was determined by gas chromatography (GC), using a similar methodology as in a previous study [18]. In short, the chloroform–methanol (1:1, *v*/*v*) method was used to extract lipids from the meat samples. KOH–methanol was used to produce fatty acid methyl esters for GC determination. The top phase, which contained fatty acid methyl esters (FAMEs), was collected and utilized in the gas chromatograph (Agilent Technologies, Santa Clara, CA, USA) equipped with a 60 μm × 0.25 mm × 0.25 μm HP-88 column and a mass detector (MSD 5977A) to separate the FA.

### 2.7. RNA Isolation and Real-Time PCR Verification

The extraction of total RNA from the samples was carried out via the Trizol approach (Invitrogen, Carlsbad, CA, USA). DNase I treatment (Takara Biotechnology Co., Ltd., Dalian, China) was applied to get rid of genomic DNA contamination. The quality and purity of the RNA were evaluated by gauging absorbance ratios (260/280 nm and 260/230 nm) with a Nanodrop—2000 spectrophotometer (Thermo Scientific, Wilmington, DE, USA). When it comes to cDNA synthesis, 1 μg of RNA treated with DNase from each sample was reverse-transcribed using 5X All-In-One RT Master Mix (Applied Biological Materials Inc., Richmond, BC, Canada). The resulting cDNA products were diluted five-fold with nuclease-free water and normalized to equivalent concentrations for subsequent PCR amplification. Gene-specific primers were designed using Primer 3 -BLAST (http://www.ncbi.nlm.nih.gov/tools/primer-blast/ accessed on 11 June 2025) based on published sequences in GenBank. All primers, which were commercially produced by Shanghai Biotechnology Company (Shanghai, China), had their sequences detailed in Table 1.

### 2.8. Statistical Analysis

An independent-samples *t*-test was employed to analyze the mean differences between HWs and XSs in terms of carcass characteristics, meat quality, and amino acid and fatty acid profiles with the use of SPSS 16.0 software (SPSS Inc., Chicago, IL, USA). Pearson correlation coefficient analysis was performed to assess the correlation between gene expression and fatty acid composition in each group. Results with a *p*-value < 0.05 were considered statistically significant.

## 3. Results

### 3.1. Comparison of Slaughter and Carcass Traits Between HW and XS

**Figure 1:**
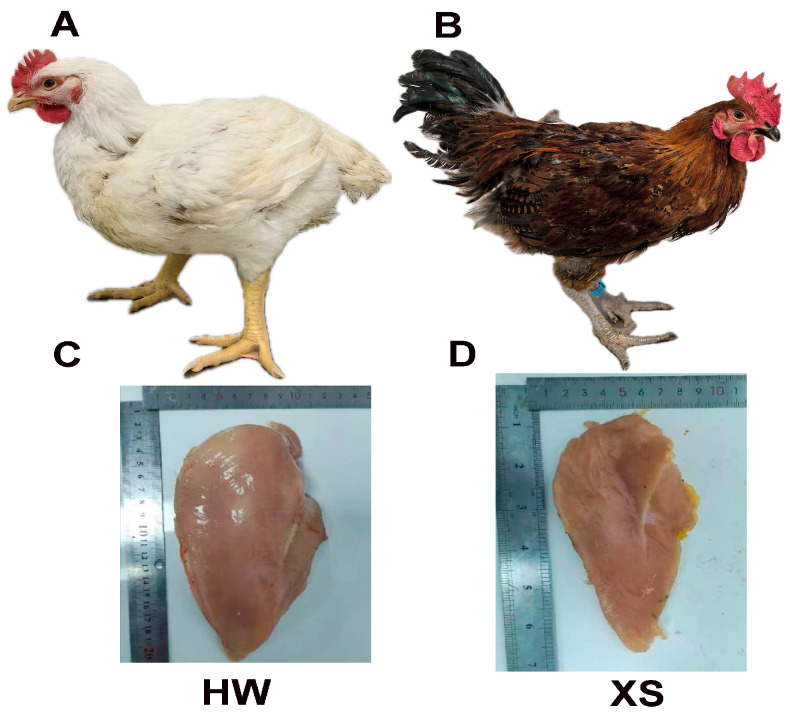
Different phenotypic characteristics between Hubbard white broilers (HWs) and Xueshan chickens (XSs). Characteristics of appearance of HW (**A**) and (**B**) XS. Breast muscle of HW (**C**) and XS (**D**).

The difference in carcass traits between HWs and XSs is shown in Figure 1. In terms of phenotype, the HW was found to have heavier weight and greater breast muscles than the XS. The comparison of slaughter performance and carcass traits of HWs and XSs are shown in Table 2. At the market age, the weight of the live body, the slaughter, semi-slaughter, wing, breast muscle, and thigh muscle of HWs were significantly higher than those of XSs (Table 2) (*p* < 0.01). On the contrary, the percentage of thigh muscle in XSs was significantly higher (*p* < 0.01) than in HWs. In addition, the abdominal fat weight of XSs was higher that of the HW (Table 2) (*p* < 0.05).

### 3.2. Comparison of Muscle Fiber Characteristics Between HW and XS

**Figure 2:**
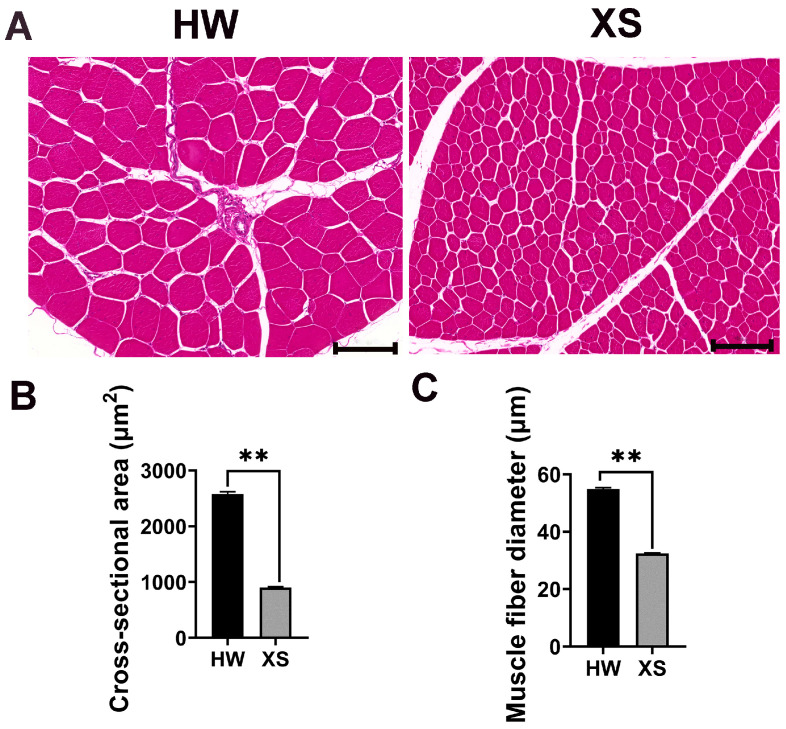
The morphology and characteristics of myofibers in HWs and XSs. (**A**) The morphology of myofibers in breast muscles of HWs and XSs was observed by HE staining. Scale bar = 100 μm. Muscle fiber cross-sectional area (**B**) and muscle fiber diameter (**C**) were analyzed. Vertical bars represent mean ± SE (*n* = 6). *p* < 0.01 is shown as **.

The morphological traits of the muscle fibers were determined by histological staining between HWs and XSs. Consistent with the results in Figure 1, HWs exhibited a larger muscle fiber area and diameter than XSs, but muscle fibers were more closely aligned in XSs than in HWs. As shown in Figure 2B, the average area of the myofiber of HW breast muscles (2578 ± 41.28 μm^2^) was significantly higher than that of XSs (902.2 ± 13.65 μm^2^) (*p* < 0.01). The diameter of the myofiber of HWs (54.91 ± 8.47 μm) was remarkably higher than that of XSs (32.41 ± 4.78 μm) (Figure 2C).

### 3.3. Comparison of Physical Characteristics Between HW and XS

The comparison of physical characteristics in the breast muscles of HWs and XSs is shown in Table 3. The results showed that the a*_24_, b*_24_, and L*_24_ values of the breast muscles of XSs were significantly higher than those of HWs (Table 3) (*p* < 0.01). The pH of the breast muscles of HWs was 6.52 ± 0.04 at 1 h, and decreased to 6.47 ± 0.05 at 24 h, whereas the pH was lower in XSs at both 1 h (6.06 ± 0.06) and 24 h (5.97 ± 0.04) compared with HWs (Table 3) (*p* < 0.01). The breast muscles of the HWs exhibited a higher shear force than did those of the XSs, indicating that XS meat is tenderer. Moreover, the drip loss of the HWs was significantly lower compared with XSs (Table 3) (*p* < 0.01). There was no significant difference in the water-holding capacity of breast muscles between HWs and XSs (*p* > 0.05).

### 3.4. Analysis of Amino Acid Profile Between HWs and XSs

To compare the composition of the breast muscles of HWs and XSs, different types of amino acids were determined using amino acid auto-analyzer systems. Compared with HWs, there was no significant difference in the amino acid composition of the breast muscles of XSs (Table 4) (*p* > 0.05). However, the XSs had a higher content of flavor amino acids (FAAs) than those of HWs, including glutamic acid (Glu), proline (Pro), alanine (Ala), serine (Ser), glycine (Gly), and Isoleucine (Ile) (*p* < 0.05).

### 3.5. Analysis of Fatty Acid Profile Between HWs and XSs

The sum of the fatty acid composition of the breast muscles of XSs and HWs was determined by gas chromatography. As shown in Table 5, the fatty acid composition was similar for both HWs and XSs. The analysis revealed that the contents of palmitic (C16:0) and myristic acid (C14:0), which represent saturated fatty acids (SFAs), were significantly higher in XSs than in HWs (Table 5) (*p* < 0.01). In addition, a higher content of arachidonic (C20:4 n-6) and docosapentaenoic (C22:6 n-3) acids, representing polyunsaturated fatty acids (PUFAs), in the breast muscles of XSs than HWs was found (*p* < 0.01). However, the content of elaidic acid (C18:1n9t), oleic acid (C18:1n9c), linoleic acid (C18:2 n-6), and cis-11, 14-Eicosadienoic acid (C20:2) representing monounsaturated fatty acids (MUFAs) were significantly lower in XSs than HWs (Table 5) (*p* < 0.01).

### 3.6. The mRNA Expression of Lipid Metabolism-Related Genes of HWs and XSs

**Figure 3:**
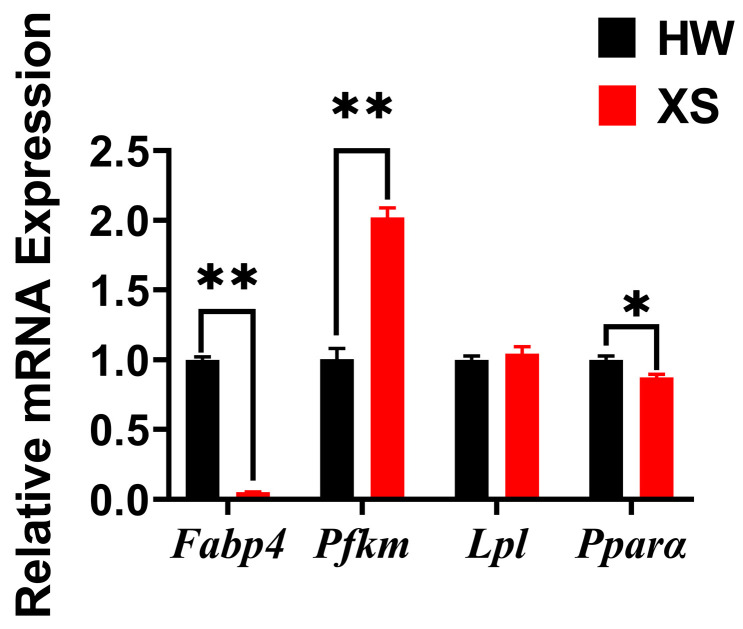
In breast muscles, the expression levels of mRNA from genes related to lipid metabolism. The mRNA expression of *Fabp4*, *Pfkm*, *Lpl*, and *Pparα* in HWs and XSs was tested by qPCR. Data are presented as the means ± SE. *p* < 0.05 is shown as *, *p* < 0.01 is shown as **.

The relative expression levels of mRNA for genes associated with lipid metabolism, such as phosphofructokinase, muscle type (*Pfkm*), peroxisome proliferator-activated receptor alpha (*Pparα*), fatty acid binding protein 4 (*Fabp4*), and lipoprotein lipase (*Lpl*) in the breast muscles of HWs and XSs were measured by qRT-PCR. The result suggested that the mRNA expression level of the *Pfkm* gene was significantly higher in XSs than in HWs (*p* < 0.01) (Figure 3). In addition, the mRNA expression of *Fabp4* and *Pparα* was significantly lower in XSs than in HWs (Figure 3). There was no significant difference in the mRNA expression of *Lpl* between HWs and XSs (*p* > 0.05).

### 3.7. Correlation Between Gene Expression and Fatty Acid Composition

**Figure 4:**
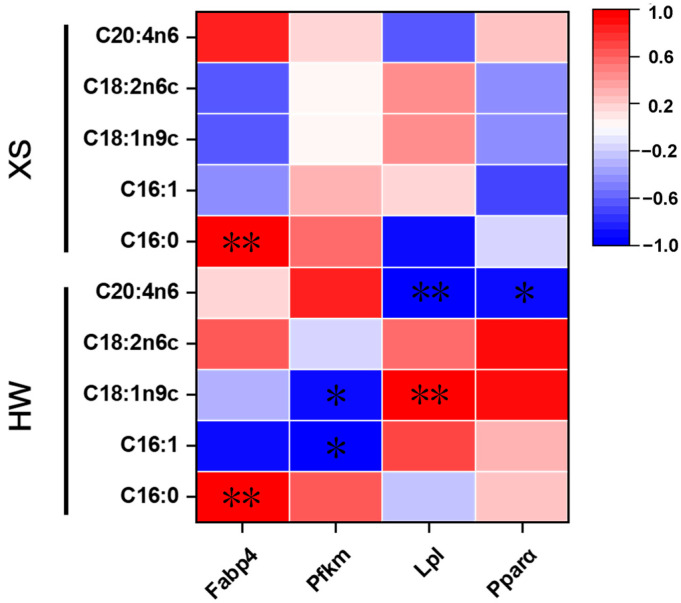
Pearson’s correlation coefficient plots. The mRNA expression levels of lipid metabolism-related genes and the fatty acid composition of HWs and XSs was analyzed. *p* < 0.05 is shown as *, *p* < 0.01 is shown as **.

Finaly, whether there is a correlation between the mRNA expression level of lipid metabolism-related genes and fatty acid composition was analyzed. The results showed that the mRNA expression of *Fabp4* was significantly positively correlated with palmitic (C16:0) in both HWs and XSs (Figure 4). In HWs, the mRNA expression of *Lpl* was significantly positively correlated with oleic acid (C18:1n9c), but negatively correlated with C20:4n6 (*p* < 0.01) (Figure 4). Furthermore, the mRNA expression of the *Pfkm* gene was negatively correlated with oleic acid (C18:1n9c) and palmitoleic acid (16:1) (*p* < 0.05) (Figure 4).

## 4. Discussion

This experiment revealed expectedly superior slaughter parameters in HWs compared to XSs. The latter, however, accumulated more abdominal fat. The higher abdominal fat (%) was observed in XSs, which may also be associated with their age. Previous studies have suggested that the breast muscle weight of commercial broilers is higher, which may be due to the fact that their breast muscles contain high concentration of insulin-like growth factor [18]. Commercial broilers showed higher breast weight than native breeds in previous observations [19,20]. The results of this study are consistent with prior research indicating a positive correlation between carcass traits and breast muscle weight. In this study, for XSs the ratio of semi-evisceration yield and thigh muscle were 81.69% and 25.93%. Thigh weight was significantly higher in XSs compared to HWs, potentially due to their higher activity levels, which promote skeletal development and muscle mass accumulation [9]. These contrasting phenotypes may reflect differential metabolic phenotypes between the two breeds.

Previous studies have indicated that, in terms of flavor, color, and tenderness, native chicken breeds are considered to possess superior meat quality compared to fast-growing broiler breeds [21,22,23,24]. In our study, significant differences between the compared chicken breeds were noted for the L*, b*, and a* colors of the breast muscles. Meat color is generally influenced by the age of the animals and their genotype [25]. With increasing age, the myoglobin content in broiler breast muscle rises [9], leading to significantly higher a* values in the XSs’ breast muscle compared to that of the HWs. The XSs were lower than HWs in terms of cooking loss and Warner–Bratzler shear force. The tenderness of the meat is the most influential factor in terms of consumer perceptions of meat palatability [7,24]. The difference in shear force values between the two chicken breeds may be related to an increase in intramuscular connective tissue with age [26]. Muscle fiber diameter and shear force have been found to significantly positively correlate in poultry [27]. Therefore, we calculated the myofiber cross-sectional area and myofiber diameter of XSs and HWs by HE staining. It was determined in this study that the higher shear force and lower drip loss in HWs compared to those in XSs might be due to the larger muscle fibers with less extracellular space in the HWs.

The nutritional quality, appearance, and flavor of poultry meat are substantially impacted by the fatty acid and amino acid profiles of the muscle tissues [28]. Native chicken breeds outperform broilers in terms of flavor and meatiness, as demonstrated by previous research [29,30]. The present study showed that HWs had a similar fatty acid composition to XSs, but that the content of each fatty acid was significantly different between HWs and XSs. The impact of fatty acids on meat quality is primarily contingent upon the fatty acid content [31]. This study shows that the content of SFAs was significantly higher in the breast muscles of the XSs than in those of HWs, and PUFA was higher in XSs. During oxidation, PUFAs in lipids break down muscle fiber bundles, which enhances meat quality. Moreover, through a series of oxidative reactions, they produce flavor-related compounds, including esters, ketones, alcohols, aldehydes, and aliphatic compounds [20,32]. In this experiment, when compared with HWs, the XSs showed higher amounts of C20:4n6 in their breast muscles, which is not only beneficial to human health but also improves the freshness of the meat flavor [33]. However, the content of MUFAs in the XSs was lower than in the HWs. Oxidized MUFAs can potentially reduce the nutritional value of meat and deteriorate its sensory quality by leading to the production of harmful substances such as free radicals [34]. MUFAs play a crucial role in maintaining water-holding capacity and modulating myofibrillar protein stability. A previous study reported differences in SFA and PUFA content between Thai indigenous chickens and commercial broilers, but no difference in MUFA content [23]. These findings indicated that the fatty acid content of XSs is better than that of HWs.

In order to investigate the molecular mechanism behind the variations in fatty acid profile between HWs and XSs, the expression levels of *Pparα*, *Pfkm*, *Lpl*, and *Fabp4* were subjected to further investigation. The process of adipocyte differentiation involves a series of changes in the regulation of differentiation transcription factors and the expression of genes regulating lipid metabolism [35]. *Lpl*, which is produced and secreted by adipocytes, regulates the concentration of triglyceride (TG) in the serum [36]. Among them, the PPAR signaling pathway is the most important pathway in terms of regulating fatty acid biosynthesis and metabolism [37]. Pparα induces the synthesis of fatty acid transporter proteins, promotes the entry of fatty acids into cells, increases lipid oxidation metabolism, and reduces triglyceride accumulation [38]. In addition, *Fabp4* exhibited higher expression levels in the breast muscles of HWs. It has been demonstrated that *Fabp4* is involved in transport of fatty acids from the cytoplasm to organelle membranes [39]. Phosphofructokinase (*Pfkm*) is a key regulatory enzyme involved in glycolysis [40]. The enzyme catalyzes the conversion of fructose-6-phosphate to fructose-1,6-bisphosphate and ADP [41]. Its expression correlates positively with Myo glycogen content and negatively with pH, and it has a direct effect on the efficiency of glycolysis [2]. The expression levels of the *Pfkm* and *Fabp4* genes could serve as a potential indicator for assessing fatty acid content.

## 5. Conclusions

In conclusion, this study revealed significant differences in carcass weight and meat quality between HW and XS at market ages. The slaughter performance traits of XSs were lower than HWs, except for the ratios of thigh muscle and abdominal fat weight, indicating that HWs had a high yield and fast growth. In contrast, the breast muscle of XSs showed more redness and yellowness, higher tenderness, as well as lower thermal loss compared to that of HWs. Moreover, the content of PUFAs was high in XSs, which is likely to produce flavor compounds while cooking. Furthermore, the expression of *Pfkm* and *Fabp4* had a close correlation with the lipid metabolism and flavor formation in chicken. Therefore, this study will provide reference data about the carcass composition and the nutritive and physicochemical properties of two chicken breeds, which could be an objective evaluation for consumers.

## Figures and Tables

**Table 1:** Primer information of qRT-PCR.

Gene	GenBank Accession	Primer Sequence (5′-3′)	Product Size (bp)
*Fabp4*	NM_204290.2	F:GATGAGACCACAGCAGATGACAG	120
		R:ATCCACCACTTTCCTCTTGATAACAG	
*Pfkm*	XM_046904942.1	F:TGTGACAACAGCGATGAATGAGAG	117
		R:GTGGAGGGTGGGCGGATG	
*Pparα*	NM_001001464.1	F:ACCAGCATCCAGTCCTTCATCC	112
		R: TGAGGCTTTATCCCCACAAATTCTAC	
*Lpl*	NM_205282.2	F:ACGGTGACAGGAATGTATGAAAGC	99
		R:AACCAGCCAGTCCACAACAATG	
*β-actin*	NM_205518.2	F:CAGCCATGTATGTAGCCATCCAG	84
		R:CATCACCAGAGTCCATCACAATACC	

**Table 2:** Comparison of Slaughter Performance between HW and XS.

Items	HW	XS	*p* Value
Live body weight (g)	3016.10 ± 37.32 ^A^	1668.82 ± 70.71 ^B^	0.000
Slaughter weight (g)	2826.69 ± 32.71 ^A^	1535.53 ± 63.45 ^B^	0.000
Semi-eviscerated weight (g)	2586.77 ± 34.71 ^A^	1357.61 ± 58.38 ^B^	0.000
Eviscerated weight (g)	2154.81 ± 34.03 ^A^	1002.17 ± 40.31 ^B^	0.000
Wing weight (g)	219.49 ± 5.17 ^A^	142.92 ± 6.71 ^B^	0.000
Breast muscle weight (g)	730.13 ± 20.27 ^A^	179.46 ± 8.28 ^B^	0.000
Thigh muscle weight (g)	487.25 ± 12.29 ^A^	259.16 ± 15.47 ^B^	0.000
Abdominal fat weight (g)	23.65 ± 2.10 ^b^	35.10 ± 4.79 ^a^	0.037
Semi-eviscerated yield (%)	85.75 ± 0.27 ^A^	81.69 ± 0.42 ^B^	0.000
Eviscerated yield (%)	71.46 ± 0.79 ^A^	61.34 ± 0.62 ^B^	0.000
Thigh muscles in carcass (%)	22.65 ± 0.57 ^B^	25.93 ± 0.67 ^A^	0.001
Breast muscles in carcass (%)	33.94 ± 0.96 ^A^	17.60 ± 0.28 ^B^	0.000

Statistically significant differences (*p* < 0.05) are indicated by a or b. Extremely statistically significant differences (*p* < 0.01) are indicated by A or B.

**Table 3:** Comparison of Physicochemical Characteristics of Breast Muscle Quality of HWs and XSs.

Items	HW	XS	*p* Value
pH	6.52 ± 0.04 ^A^	6.06 ± 0.06 ^B^	0.000
pH 24 h	6.47 ± 0.05 ^A^	5.97 ± 0.04 ^B^	0.000
L* value 24 h	27.57 ± 1.61 ^B^	45.59 ± 1.39 ^A^	0.000
a* value 24 h	2.85 ± 0.55 ^B^	6.29 ± 0.40 ^A^	0.000
b* value24h	13.01 ± 0.66 ^B^	16.68 ± 0.73 ^A^	0.000
Water-holding capacity (%)	33.36 ± 2.52	32.01 ± 2.90	0.726
Thermal loss (%)	29.01 ± 0.69 ^A^	22.78 ± 0.97 ^B^	0.000
Warner–Bratzler shear force (N)	31.10 ± 4.82 ^A^	15.48 ± 2.03 ^B^	0.000
Drip loss (%)	2.20 ± 0.18 ^B^	7.61 ± 0.47 ^A^	0.000

Extremely statistically significant differences (*p* < 0.01) are indicated by A or B.

**Table 4:** Amino Acid Composition of Breast Muscles of HWs and XSs (mg/g).

Items	HW	XS	*p* Value
Non-essential Amino Acid (NEAA)			
Glutamic acid (Glu) *	3.40 ± 0.10	3.31 ± 0.25	0.733
Aspartic acid (Asp) *	2.16 ± 0.07	2.15 ± 0.17	0.961
Arginine (Arg)	1.48 ± 0.05	1.50 ± 0.12	0.853
Alanine (Ala) *	1.39 ± 0.05	1.37 ± 0.11	0.847
Glycine (Gly) *	1.02 ± 0.03	1.09 ± 0.10	0.525
Serine (Ser)	0.87 ± 0.03	0.84 ± 0.06	0.735
Essential Amino Acid (EAA)			
Lysine (Lys)	2.12 ± 0.06	2.12 ± 0.16	0.974
Leucine (Leu)	1.88 ± 0.06	1.90 ± 0.15	0.909
Valine (Val)	1.21 ± 0.04	1.23 ± 0.10	0.852
Isoleucine (Ile)	1.16 ± 0.04	1.17 ± 0.09	0.867
Phenylalanine (Phe) *	0.92 ± 0.03	0.95 ± 0.07	0.683
Tyrosine (Tyr) *	0.79 ± 0.02	0.79 ± 0.06	0.971
Threonine (Thr)	1.02 ± 0.03	1.01 ± 0.08	0.835
Histidine (His)	0.89 ± 0.04	0.95 ± 0.07	0.482
Methionine (Met)	0.62 ± 0.02	0.62 ± 0.05	0.986
Σ EAA	11.31 ± 0.27	11.13 ± 0.21	0.615
Σ FAA	10.35 ± 0.49 ^b^	11.92 ± 0.52 ^a^	0.045

Statistically significant differences (*p* < 0.05) are indicated by a or b. Flavor amino acids are represented by *.

**Table 5:** Major Fatty Acid Composition (% of Total FA) of Breast Muscle of HWs and XSs.

Fatty Acids	HW	XS	*p* Value
C14:0	0.13 ± 0.03 ^B^	0.37 ± 0.03 ^A^	0.000
C16:0	21.94 ± 0.14 ^B^	24.68 ± 0.33 ^A^	0.000
C17:0	0.19 ± 0.01 ^a^	0.14 ± 0.02 ^b^	0.015
C18:0	9.46 ± 0.44	10.19 ± 0.50	0.219
C20:0	0.26 ± 0.12	0.14 ± 0.01	0.388
Σ SFAΣ	32.16 ± 0.56 ^B^	35.49 ± 0.61 ^A^	0.001
C16:1	2.89 ± 0.17	2.81 ± 0.40	0.850
C17:1	0.39 ± 0.05 ^B^	0.69 ± 0.08 ^A^	0.007
C18:1n9t	0.18 ± 0.01 ^A^	0.11 ± 0.01 ^B^	0.000
C18:1n9C	33.05 ± 0.55 ^A^	27.14 ± 0.99 ^B^	0.000
Σ MUFA	36.52 ± 0.66 ^A^	30.68 ± 1.28 ^B^	0.001
C18:2n6C	23.62 ± 0.46 ^A^	21.30 ± 0.43 ^B^	0.002
C18:2nt	0.09 ± 0.01	0.14 ± 0.02	0.075
C20:2	0.96 ± 0.18 ^A^	0.36 ± 0.03 ^B^	0.008
C20:3n6	0.64 ± 0.07	0.69 ± 0.06	0.581
C20:4n6	4.27 ± 0.53 ^B^	9.15 ± 1.07 ^A^	0.001
C22:6n3	0.19 ± 0.04 ^b^	0.43 ± 0.09 ^a^	0.036
Σ PUFA	29.42 ± 0.35 ^b^	32.04 ± 0.94 ^a^	0.018

Statistically significant differences (*p* < 0.05) are indicated by a and b. Extremely statistically significant differences (*p* < 0.01) are indicated by A and B.

## Data Availability

The raw data supporting the conclusions of this article will be made available by the authors on request.

## Abbreviations

The following abbreviations are used in this manuscript:

SFA	saturated fatty acids
CSA	cross-sectional area
MFD	myofiber diameter
PUFA	polyunsaturated fatty acids
TG	triglyceride

## References

[1] Kearney J. (2010). Food consumption trends and drivers. Philos. Trans. R. Soc. B Biol. Sci..

[2] England E.M., Matarneh S.K., Oliver E.M., Apaoblaza A., Scheffler T.L., Shi H., Gerrard D.E. (2016). Excess glycogen does not resolve high ultimate pH of oxidative muscle. Meat Sci..

[3] Nematbakhsh S., Selamat J., Idris L.H., Abdull Razis A.F. (2021). Chicken Authentication and Discrimination via Live Weight, Body Size, Carcass Traits, and Breast Muscle Fat Content Clustering as Affected by Breed and Sex Varieties in Malaysia. Foods.

[4] MacRae V.E., Mahon M., Gilpin S., Sandercock D.A., Mitchell M.A. (2006). Skeletal muscle fibre growth and growth associated myopathy in the domestic chicken (*Gallus domesticus*). Br. Poult. Sci..

[5] Weng K., Huo W., Li Y., Zhang Y., Zhang Y., Chen G., Xu Q. (2022). Fiber characteristics and meat quality of different muscular tissues from slow- and fast-growing broilers. Poult. Sci..

[6] Sarsenbek A., Wang T., Zhao J.K., Jiang W. (2013). Comparison of carcass yields and meat quality between Baicheng-You chickens and Arbor Acres broilers. Poult. Sci..

[7] Devatkal S.K., Naveena B.M., Kotaiah T. (2019). Quality, composition, and consumer evaluation of meat from slow-growing broilers relative to commercial broilers. Poult. Sci..

[8] Rajkumar U., Muthukumar M., Haunshi S., Niranjan M., Raju M.V., Rama Rao S.V., Chatterjee R.N. (2016). Comparative evaluation of carcass traits and meat quality in native Aseel chickens and commercial broilers. Br. Poult. Sci..

[9] Singh M., Lim A.J., Muir W.I., Groves P.J. (2021). Comparison of performance and carcass composition of a novel slow-growing crossbred broiler with fast-growing broiler for chicken meat in Australia. Poult. Sci..

[10] Devatkal S.K., Vishnuraj M.R., Kulkarni V.V., Kotaiah T. (2018). Carcass and meat quality characterization of indigenous and improved variety of chicken genotypes. Poult. Sci..

[11] Tang H., Gong Y.Z., Wu C.X., Jiang J., Wang Y., Li K. (2009). Variation of meat quality traits among five genotypes of chicken. Poult. Sci..

[12] Mussa N.J., Kibonde S.F., Boonkum W., Chankitisakul V. (2022). The Comparison between Tanzanian Indigenous (Ufipa Breed) and Commercial Broiler (Ross Chicken) Meat on the Physicochemical Characteristics, Collagen and Nucleic Acid Contents. Food Sci. Anim. Resour..

[13] Gumułka M., Połtowicz K. (2020). Comparison of carcass traits and meat quality of intensively reared geese from a Polish genetic resource flock to those of commercial hybrids. Poult. Sci..

[14] Weng K., Li Y., Huo W., Zhang Y., Cao Z., Zhang Y., Xu Q., Chen G. (2022). Comparative phosphoproteomic provides insights into meat quality differences between slow- and fast-growing broilers. Food Chem..

[15] Kokoszyński D., Żochowska-Kujawska J., Kotowicz M., Sobczak M., Piwczyński D., Stęczny K., Majrowska M., Saleh M. (2022). Carcass characteristics and selected meat quality traits from commercial broiler chickens of different origin. Anim. Sci. J..

[16] Li F., Lu Y., He Z., Yu D., Zhou J., Cao H., Zhang X., Ji H., Lv K., Yu M. (2024). Analysis of carcass traits, meat quality, amino acid and fatty acid profiles between different duck lines. Poult. Sci..

[17] Liang F., Yan L., Li Y., Jin Y., Zhang J., Che H., Diao J., Gao Y., He Z., Sun R. (2022). Effect of season on slaughter performance, meat quality, muscle amino acid and fatty acid composition, and metabolism of pheasants (*Phasianus colchicus*). Anim. Sci. J..

[18] Xiao Y., Wu C., Li K., Gui G., Zhang G., Yang H. (2017). Association of growth rate with hormone levels and myogenic gene expression profile in broilers. J. Anim. Sci. Biotechnol..

[19] Mueller S., Kreuzer M., Siegrist M., Mannale K., Messikommer R.E., Gangnat I.D.M. (2018). Carcass and meat quality of dual-purpose chickens (Lohmann Dual, Belgian Malines, Schweizerhuhn) in comparison to broiler and layer chicken types. Poult. Sci..

[20] Fanatico A.C., Pillai P.B., Hester P.Y., Falcone C., Mench J.A., Owens C.M., Emmert J.L. (2008). Performance, livability, and carcass yield of slow- and fast-growing chicken genotypes fed low-nutrient or standard diets and raised indoors or with outdoor access. Poult. Sci..

[21] Jin J., Xue M., Tang Y., Zhang L., Hu P., Hu Y., Cai D., Luo X., Sun M.A. (2023). Effects of Zinc Source and Level on the Intestinal Immunity of Xueshan Chickens under Heat Stress. Animals.

[22] Haunshi S., Devatkal S., Prince L.L.L., Ullengala R., Ramasamy K., Chatterjee R. (2022). Carcass Characteristics, Meat Quality and Nutritional Composition of Kadaknath, a Native Chicken Breed of India. Foods.

[23] Wattanachant S., Benjakul S., Ledward D.A. (2004). Composition, color, and texture of Thai indigenous and broiler chicken muscles. Poult. Sci..

[24] Mir N.A., Rafiq A., Kumar F., Singh V., Shukla V. (2017). Determinants of broiler chicken meat quality and factors affecting them: A review. J. Food Sci. Technol..

[25] Choo Y.K., Kwon H.J., Oh S.T., Um J.S., Kim B.G., Kang C.W., Lee S.K., An B.K. (2014). Comparison of growth performance, carcass characteristics and meat quality of korean local chickens and silky fowl. Asian-Australas. J. Anim. Sci..

[26] Nakamura Y.N., Iwamoto H., Shiba N., Miyachi H., Tabata S., Nishimura S. (2004). Developmental states of the collagen content, distribution and architecture in the pectoralis, iliotibialis lateralis and puboischiofemoralis muscles of male Red Cornish x New Hampshire and normal broilers. Br. Poult. Sci..

[27] Huo W., Weng K., Gu T., Zhang Y., Zhang Y., Chen G., Xu Q. (2021). Effect of muscle fiber characteristics on meat quality in fast- and slow-growing ducks. Poult. Sci..

[28] Kamal R., Chandran P.C., Dey A., Sarma K., Padhi M.K., Giri S.C., Bhatt B.P. (2022). Status of Indigenous duck and duck production system of India—A review. Trop. Anim. Health Prod..

[29] Fu Y., Cao S., Yang L., Li Z. (2022). Flavor formation based on lipid in meat and meat products: A review. J. Food Biochem..

[30] Chaiwang N., Marupanthorn K., Krutthai N., Wattanakul W., Jaturasitha S., Arjin C., Sringarm K., Setthaya P. (2023). Assessment of nucleic acid content, amino acid profile, carcass, and meat quality of Thai native chicken. Poult. Sci..

[31] Wood J.D., Enser M., Fisher A.V., Nute G.R., Richardson R.I., Sheard P.R. (1999). Manipulating meat quality and composition. Proc. Nutr. Soc..

[32] Benet I., Guàrdia M.D., Ibañez C., Solà J., Arnau J., Roura E. (2016). Low intramuscular fat (but high in PUFA) content in cooked cured pork ham decreased Maillard reaction volatiles and pleasing aroma attributes. Food Chem..

[33] Takahashi H. (2018). Association Between Arachidonic Acid and Chicken Meat and Egg Flavor, and Their Genetic Regulation. J. Poult. Sci..

[34] Calder P.C. (2015). Functional Roles of Fatty Acids and Their Effects on Human Health. J. Parenter. Enter. Nutr..

[35] Guo Y., Guo X., Deng Y., Cheng L., Hu S., Liu H., Hu J., Hu B., Li L., He H. (2020). Effects of different rearing systems on intramuscular fat content, fatty acid composition, and lipid metabolism-related genes expression in breast and thigh muscles of Nonghua ducks. Poult. Sci..

[36] Eckel R.H. (1989). Lipoprotein lipase. A multifunctional enzyme relevant to common metabolic diseases. N. Engl. J. Med..

[37] Wagner N., Wagner K.D. (2020). The Role of PPARs in Disease. Cells.

[38] Bougarne N., Weyers B., Desmet S.J., Deckers J., Ray D.W., Staels B., De Bosscher K. (2018). Molecular Actions of PPARα in Lipid Metabolism and Inflammation. Endocr. Rev..

[39] Garin-Shkolnik T., Rudich A., Hotamisligil G.S., Rubinstein M. (2014). FABP4 attenuates PPARγ and adipogenesis and is inversely correlated with PPARγ in adipose tissues. Diabetes.

[40] Wang J., Qin L., Feng Y., Zheng R., Deng C., Xiong Y., Zuo B. (2014). Molecular characterization, expression profile, and association study with meat quality traits of porcine PFKM gene. Appl. Biochem. Biotechnol..

[41] Uyeda K. (1979). Phosphofructokinase. Adv. Enzymol. Relat. Areas Mol. Biol..

